# Context-based FISH localization of genomic rearrangements within chromosome 15q11.2q13 duplicons

**DOI:** 10.1186/1755-8166-4-15

**Published:** 2011-08-08

**Authors:** Wahab A Khan, Joan HM Knoll, Peter K Rogan

**Affiliations:** 1 Department of Biochemistry, University of Western Ontario, Laboratories of Genome Bioinformatics and Genomic Disorders, 1151 Richmond Street, London, ON, Canada; 2 Department of Pathology, University of Western Ontario, Laboratories of Genome Bioinformatics and Genomic Disorders, 1151 Richmond Street, London, ON, Canada; 3 Department of Computer Science, University of Western Ontario, Laboratories of Genome Bioinformatics and Genomic Disorders, 1151 Richmond Street, London, ON, Canada

## Abstract

**Background:**

Segmental duplicons (SDs) predispose to an increased frequency of chromosomal rearrangements. These rearrangements can cause a diverse range of phenotypes due to haploinsufficiency, in *cis *positional effects or gene interruption. Genomic microarray analysis has revealed gene dosage changes adjacent to duplicons, but the high degree of similarity between duplicon sequences has confounded unequivocal assignment of chromosome breakpoints within these intervals. In this study, we localize rearrangements within duplicon-enriched regions of Angelman/Prader-Willi (AS/PWS) syndrome chromosomal deletions with fluorescence *in situ *hybridization (FISH).

**Results:**

Breakage intervals in AS deletions were localized recursively with short, coordinate-defined, single copy (SC) and low copy (LC) genomic FISH probes. These probes were initially coincident with duplicons and regions of previously reported breakage in AS/PWS. Subsequently, probes developed from adjacent genomic intervals more precisely delineated deletion breakage intervals involving genes, pseudogenes and duplicons in 15q11.2q13. The observed variability in the deletion boundaries within previously described Class I and Class II deletion AS samples is related to the local genomic architecture in this chromosomal region.

**Conclusions:**

Chromosome 15 abnormalities associated with SDs were precisely delineated at a resolution equivalent to genomic Southern analysis. This context-dependent approach can define the boundaries of chromosome rearrangements for other genomic disorders associated with SDs.

## Introduction

The human genome contains numerous regions that exhibit rare structural chromosome rearrangements due to segmental duplicons (SDs) that predispose to recurrent genomic disorders [1,2]. SDs are composed of large (10 kb-400 kb) near identical (> 95%) paralogs of DNA, that are found at physically distinct genomic locations and can include genes and pseudogenes [3]. There are at least 20 distinct genomic sites in the human genome flanked by duplicons implicated in recurrent pathogenic rearrangements [3]. Among these are deletions of chromosome 15q11.2q13 in Angelman (AS [MIM 105830]) and Prader-Willi syndromes (PWS [MIM 176270]). AS and PWS share two common size classes of *de novo *deletions that differ in proximal extent of the deletion [4]. Class I (~7 Mb) and Class II (~5 Mb) deletions have variable genomic lengths and span from proximal to distal breakpoints 1 (BP1) to 3 (BP3) and breakpoints 2 (BP2) to 3 (BP3), respectively [5,6]. Large SDs containing sequences in the *HERC2 *gene family (Hect Domain and *RLD2 *[MIM 605837]), arising by transposition to the proximal and distal ends of chromosome 15q11.2q13, have been localized to the BP1, BP2 and BP3 hotspots [7,8]. Additional genomic architectures containing *GOLGA8E*-associated SDs (golgin subfamily a, 8E) can catalyze rearrangements between 15q11 and 15q24q26 [9].

Deletions in chromosome 15q11.2q13 have been characterized with custom-designed DNA microarrays and confirmed by fluorescence *in situ *hybridization (FISH) using BAC clones [10-13]. Breakage activity within highly homologous duplicons, however, is challenging to ascertain with techniques such as array CGH alone, because interpretation of context-independent hybridization data is confounded by the presence of multiple closely related, non-contiguous SDs [14].

The aim of this study was to delineate SDs at the boundaries of 15q11.2q13 deletions by FISH using individual sequence-defined, short-target single copy (SC) and low copy (LC) DNA probes [15,16]. LC probes occur in 2 to 10 copies in the haploid genome. Genomic coordinate-defined SC-FISH has been used for diagnosis of congenital and acquired disorders [15,16], including, for example, definition of an atypical microdeletion in Smith-Magenis Syndrome (SMS [MIM 182290]) [16]. Similarly designed SC probes, composed of tiled sets of oligonucleotides, spanning larger targets have also been used to detect chromosomal abnormalities [17]. In the present study, SC and LC probes are embedded within and adjacent to SD sequences. A set of 15q11.2q13 LC probes and adjacent SC probes were developed to interrogate Class I and Class II AS deletions on metaphase chromosomes. Using sequential hybridizations of LC and SC FISH probes, it is possible to determine which duplicon intervals have been retained or have been disrupted in AS deletions.

## Methods

### Categorization of breakage of documented AS/PWS deletions

**Figure 1:**
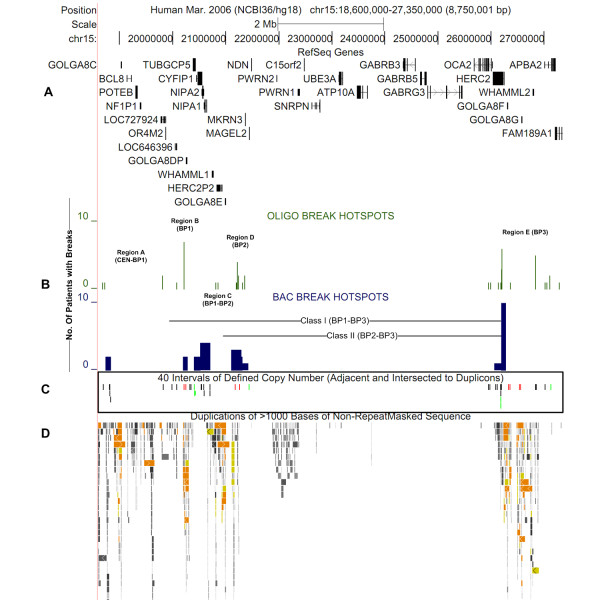
**Proximal and Distal AS/PWS Breakpoint Hotspots, Duplicon Structures, Genes, SC and LC Intervals in chromosome 15q11.2q13**. Panel A shows genomic coordinates (x-axis) and relevant genes. Panel B depicts frequency (y-axis) of previously reported BAC (blue histograms) and oligonucleotide (green histograms) microarray breakage point hotspots. Five breakage regions (A-E) and Class I and Class II deletions are indicated. Panel C shows SC and LC intervals that are coincident with the breakage hotspots. LC and SC intervals developed for FISH probes are shown in red and green, respectively [for higher resolution, see Figure 2A]. LC and SC intervals, depicted as black bars, display the density of coverage along 15q11.2q13 and can be used for further refinement of breakage activity within the AS/PWS duplicon clusters. Intervals for probe development were selected based on their proximity to high breakage densities and the multiplex information obtained from both proximal and distal breakpoints of Class I and Class II deletions. Panel D shows clusters of segmental duplicons and their relative position to genes, breakage hotspots, and LC and SC intervals. Colors refer to degree of similarity among paralogous sequences - orange is > 99%, yellow is 98-99% and gray hues are 90-98%.

Deletion boundaries in AS/PWS, determined previously by BAC [10-12] and oligonucleotide arrays [13], were reviewed and annotated using the UCSC genome browser [http://genome.ucsc.edu/, hg18 or NCBI 36 assembly]. These boundaries were approximated from both recombinant BAC sequences [18] and oligonucleotide probe genome coordinates [14] that showed reduced copy number. These data were used to locate the BP1, BP2 and BP3 breakage regions in Class I and Class II deletions [5,7,8]. Five breakage sub-intervals denoted in Figure 1 (Regions A through E) are centromeric to BP1 (CEN-BP1), within BP1, BP2, and BP3, respectively. Their corresponding genome coordinates are chr15:18,683,000-18,980,000 for region A (CEN-BP1); 18,980,000-20,385,000 for region B (BP1); 20,385,000-20,700,000 for region C (BP1-BP2); 20,700,000-21,356,000 for region D (BP2); and 25,941,000-27,286,000 for region E (BP3). Breakage intervals have also been annotated for 15q13.2q13.3 deletions within BP4 and BP5 (19) [not shown in Figure 1].

### Defining genomic SC and LC intervals

Genomic SC and LC sequences, ranging from 1500 to 5000 bp in length (per chromosome target), were batch-processed using the Galaxy metaserver http://main.g2.bx.psu.edu/[20]. The coordinates of these sequences in custom browser tracks derived from 15q11.2q13 were intersected with the approximate locations of documented breakpoints inferred from BAC and oligonucleotide array CGH [10-13]. SC and LC intervals overlapping and adjacent to these breakage intervals were prioritized for FISH probe design. These tracks were also used to determine the locations of SC and LC intervals relative to known SDs in 15q11.2q13 (Regions A-E). LC intervals lacking repetitive sequences (red) within blocks of SDs were identified and sorted from SC intervals (green) that mapped adjacent to duplicon structures (Figure 1C). The presence of these SC and LC intervals was confirmed in the Reference and alternate (HuRef, Celera) genome assemblies. BLAST analysis showed 100% sequence identity of SC intervals and 90-99% identity of paralogous LC intervals among the different assemblies.

**Figure 2:**
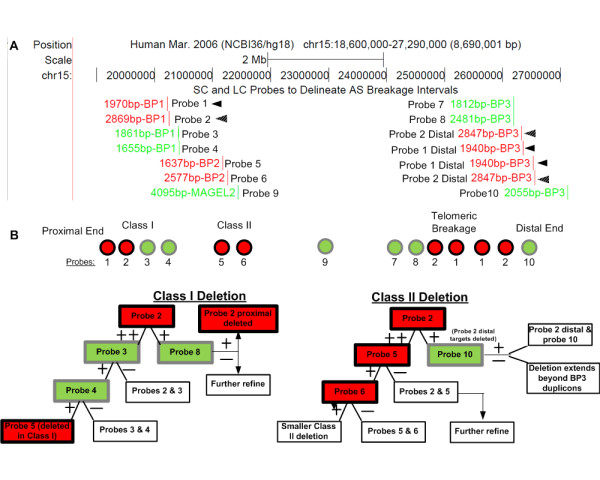
**SC and LC Probes Developed for FISH**. A) High resolution map of LC (red) and SC (green) probes. Proximal targets of probes 1 and 2 (arrowheads) in BP1 share 99% sequence identity to their distal BP3 targets (arrowheads). Probes 3, 4, 7, 8 and 10 are adjacent to duplicon regions. LC probes 5 and 6 mimic SC probes as each have one paralog target in BP2 and their other paralogs are divergent and distal of 15q11.2q13 or interchromosomal (see text). Probe 9 serves as a deletion control that targets a sequence ~20 Kb centromeric of *MAGEL2*. Probe lengths are reported in base pairs (bp). B) Metaphase FISH algorithm for delineating Class I and Class II deletions in 15q11.2q13. Probes 1 through 10 are color-coded. A schema of hybridization experiments is shown for Class I and II deletions. Different outcomes indicated by the presence (+) or absence (-) of an SC (green) or LC probe (red) prescribes the procedure for delineating boundaries of a breakage interval (unfilled box). If two paralogous LC targets are retained; this is indicated by a '++' symbol. Further refinement of a deletion interval requires either sequential application of additional SC probes or dual-color/dual-probe hybridization.

As copy number variants (CNVs) bracketed by duplicon structures can confound interpretation of FISH data, we selected only those probes with the highest sequence identity for their genomic locus and observed in the expected copy number (Figure 2A). The majority of CNVs in this region (typically > 1 Mb in BP1, BP2 and BP3) completely overlapped both SC and LC probe sequences. The presence of a polymorphic duplication separated by large genomic distances (> 5-6 Mb), coincident with SC or LC probes, in an AS deletion is expected to result in a non-contiguous hybridization pattern. There was no evidence of this pattern in our results, which is not surprising in light of the low frequency of these CNVs in the population [21]. Nevertheless, the presence of CNVs in the 15q11.2q13 region associated with SDs should be considered in genomic coordinate-defined SC and LC probe designs.

### Probe development and fluorescence *in situ *hybridization (FISH)

Primer3 [http://frodo.wi.mit.edu/primer3/[22] was used to design oligonucleotide primer pairs for 6 SC and 4 LC intervals of which one SC probe was from the common deletion region and served as a positive control (Additional File 1, Table S1). SC and LC genomic intervals were amplified using long PCR [23] with the Platinum^® ^*Pfx *DNA polymerase kit (Invitrogen™ CA, USA). PCR conditions were optimized by gradient thermal cycling. Amplicons were purified using the QIAquick gel extraction kit (Qiagen ON, Canada), labeled by nick translation with biotin-dUTP or digoxigenin-dUTP (Roche Diagnostics, ON, Canada) and detected with avidin-FITC or Cy3-conjugated digoxin antibody [24]. Probes were validated on metaphase chromosomes from at least 2 normal lymphocyte cytogenetic preparations (including one male) following approval by the Office of Research Ethics at the University of Western Ontario. Probes were analysed for chromosome location and hybridization pattern as deduced from the Human Genome Reference sequences. At least 20 metaphase cells were scored for each probe and a hybridization efficiency of ≥ 80% was required to qualify a probe.

### AS cell lines

Six AS lymphoblastoid cell lines were characterized in this study. The cell lines were previously determined to have either Class I (WJK36, WJK67, WJK70) or Class II deletions (WJK18, WJK24, WJK35) by DNA analysis [5]. The cell lines were blind coded until FISH analysis was complete. FISH with SC and LC probes were performed with either one probe/one color or two probe/two color detection to delimit the boundaries of the rearrangement within or adjacent to15q11.2q13 duplicons. Twenty to 50 metaphase cells were examined per probe for each AS cell line. Cells were imaged using an automated epifluorescence microscopy system (Metasystems Inc, MA).

## Results

### Selected SC and LC probes and their relationship to genomic architecture

Our strategy selected LC FISH probes within duplicons to interrogate rearrangements at both ends of chromosome 15q11.2q13. SC probes, adjacent to either the proximal or distal duplicons targeted by an LC probe, were then hybridized to establish whether the proximal or distal LC target was deleted. LC intervals selected for probe development were based on: 1) their location in or adjacent to a region of frequent documented breakage; 2) up to 3 chromosome 15q11.2q13 targets detected by the LC probe; and 3) genomic separation of ≥ 5 Mb for at least 2 of 3 LC probe targets with chromosome 15q11.2q13. LC probe targets separated by less than 5 Mb could not be unequivocally discriminated by FISH as distinct loci on metaphase chromosomes. Hybridization patterns of LC probes with one target within 15q11.2q13 and diverse sequence targets elsewhere in the genome were scored in a similar manner to an SC probe.

After bioinformatic analysis, 40 SC and LC intervals were marked for potential development of FISH probe reagents (Figure 1C). Nine of these intervals were selected based on the algorithm described below. They comprised 4 LC and 5 SC regions (see Figure 2A for probe map and designation). Probe details (centromere to telomere) are described below. LC probes 1 and 2 are embedded within duplicons common to BP1 and BP3. Both LC probe intervals in BP1 (probe 1, 1970 bp; and probe 2, 2869 bp) are coincident with breakage sites inferred from oligonucleotide arrays [13] and proximal to breakage sites inferred from BAC microarrays [10-12]. The LC probe intervals in BP3 (probe 1, 1940 bp; and probe 2, 2837 bp) are centrally located in the distal region of highest documented breakage activity (Figure 1B, C). SC probes were developed from within BP1 (probe 3, 1861 bp; probe 4, 1655 bp) and within BP3 (probe 7, 1812 bp; probe 8, 2481 bp). Probes 3 and 4 are adjacent to the duplicons in BP1 that are detected by LC probes 1 and 2. SC probes 7 and 8 are adjacent to duplicons in BP3 that contain probe 1 and 2 paralogs. These SC probes respectively mark the breakage boundaries within BP1 and BP3. The LC intervals in BP2 (probe 5, 1637 bp; and probe 6, 2577 bp) have homology to duplicons in 15q14 and 13q31.3, with approximately 90% sequence identity over intervals less than 1500 bp in length. The degree of sequence divergence and sizes of these paralogous targets limit the detection of hybridization of probes 5 and 6 to the 15q14 and 13q31.3 loci. Therefore, LC probes 5 and 6 mimic the hybridization patterns of SC probes in the BP2 region. SC probe 10 (2055 bp) hybridizes to a target from within *APBA2 *[MIM 602712]) which is distal of BP3, and detects larger deletions [19]. SC probe 9 (4095 bp) is a positive control for AS/PWS deletions and maps ~20 Kb centromeric of *MAGEL2 *[MIM 605283]).

### FISH algorithm

**Figure 3:**
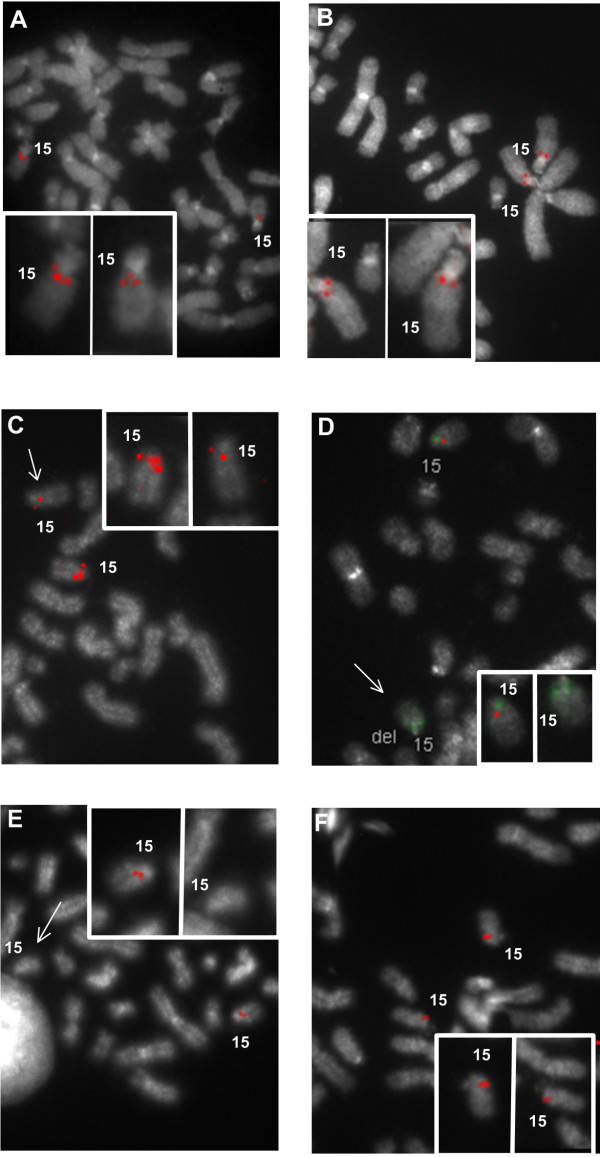
**Representative metaphase cell FISH images of SC and LC probes**. A) Hybridization pattern in a normal cell of LC probe 2 with targets in BP1 (2869 bp) and BP3 (2837 bp) to both chromosome 15s. B) Hybridization of LC probe 5 (1637 bp) on normal chromosomes. The pattern is similar to that of an SC probe, as the paralogous target of probe 5 exhibits high sequence divergence and is not detected. The next 4 panels show chromosome hybridizations on cells of Class I (C, D) and Class II (E, F) AS deletions. C) LC probe 2 hybridization pattern in WJK36 cell. Only the BP3 target is deleted in the abnormal chromosome (arrow), as determined by subsequent hybridization with SC probe 4 (not shown). The normal chromosome shows hybridization to all paralogous targets. Loss of the BP3 target sequence with this probe was also evident in WJK 70 (Class I), WJK18 and WJK24 (Class II). D) Dual-color hybridization with LC Probe 2 (green) and SC probe 3 (1861 bp, red) in WJK67 (Class I). Probe 2 is intact on both chromosomes and was confirmed by sequential hybridization. Probe 3 is deleted on the abnormal homolog (arrow), and a similar outcome for this probe was noted for WJK70. E) Deletion of SC probe 7 (1812 bp) (arrow) in WJK35. Deletions of probe 7 were also seen in WJK67, WJK70 and WJK36. F) SC probe 10 (2055 bp) is intact in WJK24. Similar hybridization patterns were seen in WJK70, WJK36 and WJK18. All probes were labeled with digoxigenin or biotin- dUTP and detected with Cy-3 digoxin antibody or FITC-avidin, respectively. Cells are counterstained with DAPI.

FISH studies with SC and LC probes validated the bioinformatic analysis. These probes can delineate boundaries of a chromosomal rearrangement in a single hybridization for certain breakpoints, however improved chromosome resolution was achieved through recursive hybridization using a series of probes. LC probes that detect fewer duplicon targets than anticipated from the genomic architecture; represent a deletion of one or more paralogs. Subsequent FISH analysis with adjacent SC probes from BP1 or BP3 can then determine which of the LC probe targets has been deleted. The process can be expedited by co-hybridizing the initial LC probe with a differentially-labeled SC probe adjacent to one of the duplicons. Figure 2B illustrates the strategy in which individual SC and LC probes are selected for FISH-based assays. The initial probe selected for hybridization varies depending upon whether the deletion has been previously classified. The order of probes used for subsequent hybridizations depends on the results of the previous FISH experiment. Both Class I and II deletions were analyzed with LC probe 2, since it targets a larger genomic interval than LC probe 1, and provides information about sequences found at both ends of the 15q11.2q13 deletion. Figure 3A shows that probe 2 hybridizes to separate duplicons in BP1 (chr15:20,241,611-20,244,479) and BP3 (chr15:26,321,705-26,324,542 and 26,550,044-26,552,881) in normal chromosomes. In AS, duplicon targets within BP1 or BP3 may be deleted. Class I breakage intervals are localized with probes 3 (proximal), 7, 8 and 10 (distal; Figure 3E and 3F). In Class II deletions, probes 5 and 10 are used for the analysis. If necessary, the Class I breakpoint can be further refined with probes 3 and 4, and Class II cases with probes 5 (Figure 3B) and 6.

### Definition of breakage intervals in AS cell lines

Prior to undertaking this study, breakage intervals at the ends of the deletions in these AS cell lines were not precisely known, and the rearranged genes and SD features coincident with deletion boundaries had not been characterized.

#### Class I deletion characterization

**Figure 4:**
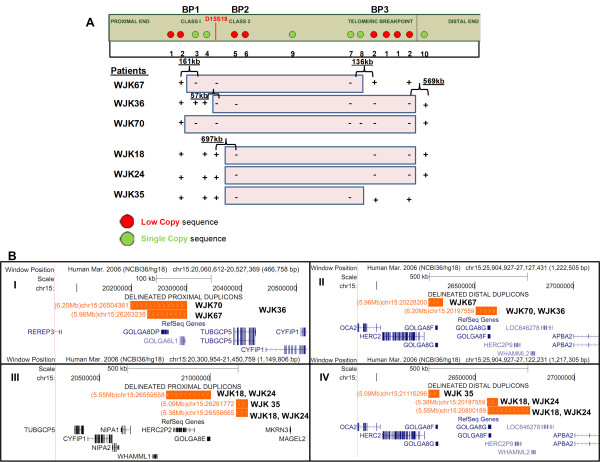
**Schematic of Proximal and Distal Breakage Intervals, Segmental Duplicons, and Genes in Class I and Class II AS Deletions**. A) Breakage intervals exhibit variability at the proximal end in Class I deletions (BP1: WJK67, WJK36, WJK70) and uniformity at the proximal end in Class II deletions (BP2: WJK18, WJK24, WJK35). Breakage intervals at the distal ends (BP3) show heterogeneity within both Class I and Class II deletions. The symbols '+' and '-' indicate the probe is intact or deleted on the abnormal homolog. Breakage interval sizes are indicated in kilobases (Kb). D15S18 is a marker that is deleted in Class I and intact in Class II AS cases [5]. B) Distinct duplicon blocks at the proximal (B-1) and distal (B-II) ends of the deletion are arranged in inverted orientation, and are separated by 5.96 Mb and 6.20 Mb in Class I patient samples WJK67 and WJK70. The proximal breakage interval for WJK36 does not involve a duplicon region. Panels B-III and B-IV: Class II patient samples, WJK18 and WJK24, overlap two pairs of duplicon blocks in the same orientation on the antisense DNA strand (< <) at the proximal and distal ends and are separated by 5.55 Mb and 5.38 Mb. WJK35 overlaps duplicon blocks separated by 5.09 Mb and are in the same orientation on the sense DNA strand (> >). In each panel (top to bottom), the genomic positions of paralogous segmental duplicons with their intrachromosomal distances (Mb) (orange), and genes (including isoforms) that are disrupted or deleted in the breakage intervals (blue or black) are indicated.

Variability in both the proximal and distal breakage intervals were observed in the three Class I AS cell lines (Figure 4A) such that each deletion differed in size. Deletion of BP3 targets in WJK36 and WJK70 resulted in single locus hybridization to the BP1 target (Figure 3C), whereas WJK67 was not deleted for either of these targets. The proximal breakage interval was localized within a 161 Kb interval in WJK67 and WJK70 (Figure 3D; between probes 2 and 3; chr15:20,244,480-20,405,122). The distal breakpoint in BP3 of WJK67 was delimited by a 136 Kb region bounded by probe 7 and one of the probe 2 duplicon targets (chr15:26,184,974-26,321,705, Figure 4A). In WJK36, the proximal breakage interval in BP1 was defined within a 57.6 Kb interval (spanning chr15:20,419,289-20,476,942), based on results showing probes 3 and 4 to be intact (Figure 4A) and a previously demonstrated deletion of D15S18 [5]. The lengths of the Class I deletions were approximately 5.78 Mb (WJK67), 6.07 Mb (WJK36), and 6.14 Mb (WJK70).

#### Class II deletion characterization

The deletion breakage interval in BP3 was found to vary among different AS cell lines, with an overall distribution similar to those observed for Class I deletions (Figure 4A). At the centromeric end, chromosomal breaks in Class II AS deletions coincide with a cluster of duplicons in BP2. Based on hybridization results using probes 4 and 5, all cell lines (WJK18, WJK24, and WJK35) exhibited a common proximal deletion breakage interval (Figure 4A). This is consistent with previous microarray analyses indicating that the probe 5 sequence maps to a highly active region of rearrangement (Figure 1B, C). The proximal breakage interval is localized to a ~697.3 Kb region within BP2, since D15S18 is intact [5] in these cell lines (chr15:20,477,088-21,174,481). The distal BP3 breakage interval in WJK35 (Class II) is the same as the one defined in WJK67 (Class I) localized by a deletion of SC probe 7 (Figure 3E). WJK18 and WJK24 shared the same breakage interval in BP3 that was present in WJK70 and WJK36. Probe 10 was intact in cell lines WJK18, WJK24, WJK70, and WJK36 (Figure 3F), thereby refining the breakage interval to a 569 Kb region (chr15:26,552,881-27,122,231;Figure 4A). The Class II deletions ranged in size from 5.01(WJK35) to 5.38 Mb (WJK18 and WJK24).

## Discussion

We demonstrate that chromosome 15q11.2q13 deletions can be delineated using combinations of single and low-copy, sequence-defined FISH probes. The LC probes detect SDs, which are prone to rearrangement. Our analysis of these rearrangements distinguishes genes and pseudogenes at the boundaries of deletions that are either deleted or disrupted in AS.

### Duplicon architecture of Class I and II AS deletions

In this study, Class I and Class II breakage intervals coincide with *HERC2*-containing SDs [7,11,25]. The proximal (103.6 Kb; coordinates: 20.2-20.3 Mb, BP1) and distal SDs (106 Kb; coordinates: 26.5-26.72 Mb, BP3) coincident with breakage intervals in WJK70 (Class I) are distinct and inversely oriented to the paralogous SDs that define breakage intervals in Class I WJK67 (Figure 4B-I, 4B-II). By contrast, in WJK36 (Class I), a more complex pattern was found. The proximal breakage interval was adjacent to the BP1 duplicons, rather than within them (Figure 4B-I). Atypical breakage intervals that fall outside of the duplicon blocks have been reported both in SMS and 16p11.2p12.2 deletions [26]. The breakage intervals in BP3 for WJK36 and WJK70 overlap the same duplicon (Figure 4B-II). Distinct blocks of SDs in BP2 mediate Class II deletion rearrangements (Figure 4B-III), which are respectively paralogous to different sets of duplicon blocks within BP3 (Figure 4B-IV). In each cell line, the proximal and distal duplicons at each end of the deletion shared 98% - 99% sequence identity.

We and others have found that 15q11.2q13 duplicons and breakage intervals in AS are in some instances coincident [12]. However, this was not the case for WJK67, WJK36 and WJK35, where breakage intervals (Figure 4A) were distinct from previously reported SDs [12]. The proximal duplicons that are rearranged in these individuals are comprised of pericentromeric *HERC2 *pseudogene sequences and the distal copy contains both the *HERC2 *gene and additional *HERC2 *pseudogenes [8,27]. Apparent non-allelic homologous recombination between these *HERC2*-related duplicons results in diverse breakage intervals with variable length deletions.

Based on the results of this study, the present FISH strategy can be further streamlined. The proximal breakage interval within Class I deletion samples can be delineated by co-hybridization with LC probe 2 and SC probe 3. Deletion of the BP3 duplicon interval can be simultaneously detected with probe 2. To expedite refinement of Class II deletion breaks, probe 5 in the BP2 duplicon cluster is co-hybridized with probe 8 in BP3. The combination of probes 4 (BP1) and 5 (BP2) can delineate the deletion class when it is unknown. A Class I deletion is indicated if both of these probes are hemizygous. A Class II deletion is diagnosed if probe 4 is retained and probe 5 is deleted. The additional SC intervals (n = 40; Figure 1C) we have designed can be used to refine the breakage sites within BP1 and BP2.

### Relating breakage intervals to genes

Contextual mapping of 15q11.2q13 genomic rearrangements bracketed by SDs can distinguish genes that are disrupted from those that are have been demonstrated to be deleted using methods that quantify copy number, i.e. array CGH, qPCR, or MLPA. *CYFIP1 *[MIM 606322] was deleted (Figure 4B-I) and a partial deletion of *TUBGCP5 *was likely (tubulin, gamma complex associated protein 5 [MIM 608147]) in WJK67 and WJK70. In contrast, the 57 Kb breakage interval of WJK36 overlapped the 3' boundary of the *TUBGCP5 *and the 5' region of *CYFIP1*, effectively disrupting both genes (Figure 4B-I). *TUBGCP5 *maps between BP1 and BP2 and encodes a protein that is required for microtubule nucleation at the centrosome [28,29]. *CYFIP1 *is associated with *FMRP *[30], which is implicated in neurite extension, guidance and branching [31]. *NIPA2 *[MIM 608146]) and *NIPA1 *[MIM 608145]), which do not overlap any duplicons, were deleted in all Class I cell lines. Dominant mutations in *NIPA1 *cause hereditary spastic paraplegia [32].

The golgin subfamily genes, *GOLGA8DP *(golgi autoantigen, golgin subfamily a, 8D) and *GOLGA6L1 *(golgi autoantigen, golgin subfamily a, 6-like 1), are embedded within SDs. Both of these genes are likely to be disrupted or deleted in WJK67 and WJK70, but are intact in WJK36 (Figure 4B-1). *GOLGA6L1 *is expressed and predicted to encode a functional protein, whereas *GOLGA8DP *is currently designated as a pseudogene [33] and has paralogy to duplicons at 15q11.2q13, 15q24 and 15q26 [9,25]. In the Class II deletions - WJK18, WJK24 and WJK35, the breakage interval in BP2 coincides with *HERC2P *(Hect domain and RLD2 pseudogene 2) and the *GOLGA8E *gene. Both *GOLGA8E *and *HERC2P *in BP2 are likely to be disrupted (Figure 4B-III). *GOLGA8E *is expressed and encodes cDNAs with an open reading frame [9].

Breakage intervals delineated in WJK67 and WJK35 overlap *HERC2*, whereas these intervals are telomeric in the other AS cell lines. This results in the deletion of *HERC2*, as well as potential deletion or disruption of *GOLGA8G, GOLGA8F *and *WHAMML2 *(Figure 4B-II, 4D-IV). *GOLGA8G *and *GOLGA8F *are expressed pseudogenes [33]. Disruption of functional *HERC2 *in BP3 can juxtapose it with distal *HERC2*-related sequences, producing novel fusion transcripts [27]. As expected, *OCA2 *([MIM 611409]) was deleted in all cell lines [5,34,35] and *APBA2*, which is distal to the telomeric *HERC2 *cluster in BP3, was intact. Large AS deletions that include *APBA2 *in 15q13.1 have been reported [12] and a small duplication of 15q13.1 has been described in a family with a history of autism [36].

### Sequence-defined FISH in other duplicon-rich genomic domains

Sequence-defined SC and LC FISH probes will be generally useful to delineate genomic disorders bracketed by SDs that juxtapose during chromosome rearrangement. In AS/PWS, SMS, and DiGeorge syndromes, the distribution of SDs enables LC probes to be designed that simultaneously interrogate sequence copy number at both ends of the chromosomal deletion. However, as seen in 15q11.2q13, some SDs may contain remote paralogous targets that do not participate in the rearrangement. These intervals can still be useful as LC probes, with the remote paralogs serving as hybridization controls in metaphase FISH.

Recently, recurrent microdeletions have been described in chromosomes 16p11.2p12.2 (chr16: 21.3-29.5 Mb) [26,37], 1q21.1 (chr1: 144.10-144.60 Mb), and a reciprocal deletion/duplication in 3q29 [Ref. [3] for review]. At the centromeric and telomeric breakpoints within 16p11.2 and 16p12.2 respectively, we identified 3 LC and 5 SC intervals, suitable as FISH probes, co-localizing with breakage activity in this region [26,37]. By combining these SC probes with different sets of centromeric LC probes, the boundaries of 16p11.2p12.2 rearrangements can be defined.

At the chromosome 1q21.1 and 3q29 loci [3], our analysis showed that duplicons flanking the common deletion interval are too closely spaced to resolve as separate hybridization signals on metaphase chromosomes. Nevertheless, these duplicon blocks can still be assayed by metaphase FISH with LC probes detecting a second interchromosomal or remote intrachromosomal locus. Generally, SC and LC FISH probes can be employed either individually or as an ensemble to analyze both large (> 5 Mb) and small (0.5 Kb) recurrent genomic rearrangements flanked by SDs. The increased resolution of SC and LC FISH in complex duplicon genomic regions may prove useful in distinguishing rearrangements that appear to be similar in length based on array CGH analysis, which actually span overlapping sequences that vary in length.

Interphase SC and LC FISH analysis may be feasible, if multiple, closely-spaced duplicon-related signals from an LC probe originate from the same homolog. We have not yet tested this possibility, given the highly decondensed state of interphase chromatin and the limited knowledge about spatial organization of homologous SDs during interphase.

### Comparison with array CGH in duplicon-rich genomic intervals

Breakage intervals of chromosomal rearrangements associated with SDs have been refined with several array CGH platforms [38-41]. BAC arrays identify the initial aberration and are refined by 2 or more customized oligonucleotide arrays. Breakpoint junctions are then delineated by long PCR or real time PCR with multiple primer pairs staggered along a duplicon block with at least one member of each pair lying in a repeat-masked sequence. Most customized oligonucleotide arrays require at least five adjacent oligonucleotide probes to be deleted or duplicated for reliable genotyping [42]. In regions containing a high degree of homology between duplicons, this strategy can result in false-positive signals from probes identical to those within the true microdeletion or microduplication [39].

Therefore, microarray designs generally avoid or provide low probe coverage in intervals that have genomic architectures such as SDs that confound the interpretation of copy number differences [43,44]. In relating the breakage intervals to the duplicon architecture and genes, we noted probes in the Agilent SurePrint 244K microarray [13] (Figure 1B) that were present in duplicons with paralogous sequences on other chromosomes and within segmentally duplicated sequences in the BP1, BP2, and BP3 regions (Table 1). On chromosome 15, the cross-hybridizing oligonucleotide probes are on average ~20 kb apart and are organized in clusters which coincide with SDs in BP1, BP2 and BP3. Since these probe sequences had been expected to be unique, their hybridization to interchromosomal and remote intrachromosomal duplicons would be expected to distort the interpretation of chromosomal deletion boundaries.

**Table 1:** Number of Agilent 244 K microarray oligonucleotide probe sets within 15q11.2q13.1 targeting distinct interchromosomal duplicons

No. of Probes	Interchromosomal Duplicon Matches	Chromosome Location
**BP1 (chr15:18,980,000-20,385,000, NCBI 36; hg18)**		

4	1	13q13.2

2	1	2q24.3

13	29	13q11, 18p11.21, 21q11.2, 2q21.1, 22q11.1, 14q11.1

3	3	17q11.2

1	1	2q14.1

11	1	14q11.2

6	1	14q32.33

1	1	Yp11.32

2	1	16q24.2

1	4	16p11.2, 13q31.3

2	1	3p22.1

1	1	20q13.12

**BP2 (chr15:20,700,000-21,356,000, NCBI 36; hg18)**		

3	1	16p13.12

1	1	13q31.3

1	1	16q12.2

**BP3 (chr15:25,941,000-27,286,000, NCBI 36; hg18)**		

3	4	16p11.2, 13q31.3

1	1	3p22.3

1	1	5p15.31

The track features from the UCSC genome browser (hg18) of the Agilent SurePrint G3 Human CGH Microarray were analyzed. The Galaxy metaserver was used to extract probe sequences coincident with the non-repeat-masked regions within BP1, BP2 and BP3. Interchromosomal duplicons with complete overlap to the Agilent probe sets were enumerated. In BP1, approximately 73% (n = 47/64) of the probes (left column) targeted distinct interchromosomal duplicon loci (middle column) in a chromosome band (right column). Probe targets in intrachromosomal duplicons and repeat-masked sequences were poorly represented in this region (27%). In BP2, approximately 74% (n = 14/19) of the Agilent 244 K probe sets targeted repeat-masked sequences or intrachromosomal duplicons; however their paralogs were distal to the BP3 region common to AS/PWS deletions. The remaining 26% (n = 5) of the probe sets targeted interchromosomal loci. Probe coverage in BP3 constituted 94% (n = 74/79) of both repeat-masked and intrachromosomal duplicon sequences. The remaining probe coverage (6%) in BP3 contained targets to both interchromosomal and intrachromosomal duplicon loci that are coincident with BP2.

The AS breakage intervals were also compared to the corresponding probe coverage by the Affymetrix SNP 6.0 microarray. Generally, there is a paucity of SNP probes within SDs. The breakage intervals delineated by SC and LC probes in the present study are not covered by any probes on the SNP array. However, several divergent SDs within BP1, BP2, and BP3 contained 3-10 probes targeting SNPs within duplicons. These probes also overlapped interchromosomal duplicons (Table 2). We further examined the subset of these SNP probes (n = 14) representing the highest degree of cross-hybridization to 15q11.2q13 breakpoint hotspots and found that 6 contained the same polymorphic variant targeting intra or interchromosmal SDs with 96-100% similarity to one another.

**Table 2:** Number of Affymetrix SNP 6.0 microarray probe sets within 15q11.2q13.1 targeting distinct interchromosomal duplicons

No. of Probes	Interchromosomal Duplicon Matches	Chromosome Location
**BP1 (chr15:18,980,000-20,385,000, NCBI 36)**		

1	1	13q13.2

3	1	2q24.3

1	1	3q29

2	3	22q12.2, 13q12.1

4	7	13q12.1, 18p11.21, 21q11.2, 2q21.1, 22q11.1, 14q11.1

2	14	18p11.2, 21q11.2, 2q21.1, 22q11.1, 14q11.1

1	1	17q11.2

1	1	2q14.1

4	1	14q11.2

1	1	12p13.31

5	2	16q24.2

2	4	1p36.23, Yq11.22

3	1	2p24.3

3	1	3p22.1

7	2	20q13.12

**BP2 (chr15:20,700,000-21,356,000, NCBI 36)**		

8	2	13q31.3

3	1	16p13.12

3	1	16p13.12

**BP3 (chr15:25,941,000-27,286,000, NCBI 36)**		

4	8	13q31.3, 16p11.2

The probe distribution along 15q11.2 q13 using the Affymetrix SNP 6.0 microarray showed 75% (n = 40/53) of the probes (left column) in BP1 occur within distinct interchromosomal duplicon loci (middle column) in a given chromosome band (right column). Probes within intrachromosomal duplicons and repeat-masked sequences showed poor representation in this region. Of the 59 probe features targeting SNPs in BP2, 19% (n = 11/59) were homologous with interchromosomal duplicon intervals and would be expected to cross-hybridize. The remaining 81% (n = 48/59) of probe features targeted repeat-masked sequences in intrachromosomal duplicons. Similarly in BP3, the majority of probes occurred within the repeat-masked sequences (89%, n = 158/177), however the remaining probes (11%) in BP3 contained interchromosomal and intrachromosomal duplicon targets. Probes within BP2 and BP3 are underrepresented relative to adjacent single copy regions.

The Illumina Beadchip (Human WG) also showed uneven coverage in the 15q11.2q13 region (Table 3). The probes did not cover BP2 at all. BP1 and BP3 were sparsely covered, with 20 probes occurring in BP1-associated SDs and 10 probes in BP3 SDs. This characteristic appears to have been intended in the array design, as poor sensitivity for detection of pathogenic CNVs associated with SDs has been noted [45,46]. Subsequent custom array CGH or MLPA were required to more precisely define these abnormalities. Of those probes detecting these intervals, the majority (19/20 in BP1 and 6/10 in BP3) of these sequences are present in multiple intra or interchromosomal duplicons.

**Table 3:** Number of Illumina Beadchip (Human WG) microarray probe sets within 15q11.2q13.1 targeting distinct interchromosomal duplicons

No. Of Probes	Interchromosomal Duplicon Matches	Chromosome Location
**BP1 (chr15:20,719,986-22,833,559, GRCh37; hg19)**		

1	1	16q12.2

1	1	13q13.3

7	3	14q32.33

1	1	21q11.2, 13q11, 18p11.21

1	6	22q11.1, 2q21.1, 18p11.21, 21q11.2, 14q11.2

1	1	16q24.2

2	1	16q12.2

1	1	20q13.12

**BP2 (chr15:23,148,559-23,804,907, GRCh37; hg19)**		

No probe coverage		

**BP3 (chr15:28,267,405-29,498,708, GRCh37; hg19)**		

3	11	16p11.2, 22q11.22, 19p13.3, 1q21.1, 22q11.21, 1q41, 13q31.3

2	1	20p12.1

Probe distribution along 15q11.2q13 with the Illumina Beadchip human whole genome array shows a high proportion of cross-hybridizing probes to SDs in BP1 and BP3, relative to those detecting unique intervals. 75% (n = 14/20) of the probes (left column) in BP1 also detect distinct interchromosomal duplicon loci (middle column) in a given chromosome band (right column). In BP3, 50% (n = 5/10) of the probes hybridized to interchromosomal targets. The remaining probes were found at paralogous loci on chromosome 15 or within repeat-masked regions. Probes within intrachromosomal duplicons (n = 5) and repeat-masked sequences (n = 1) were poorly represented in BP1 and none were found in BP2.

Copy number assessments in regions with two or more highly homologous duplicons can be incorrect due to intrinsic differences relative to balanced copy number. This affects normalization by reducing the dynamic range which can result in misinterpretations when investigating genomic copy number profiles [43,47]. This may explain some of the differences between the breakage intervals delineated in our study and those delineated by BAC and oligonucleotide microarrays. SC-FISH may also compliment genome-wide resequencing in SD-rich regions, which are more likely to contain ambiguously mapped reads [48].

## Summary

The chromosomal positions and orientations of genomic probes are essential for defining rearrangements involving SDs. Delineating these rearrangements by SC and LC FISH demonstrates disrupted genes or those whose expression is altered. For disrupted genes whose functions are associated with established phenotypes, the strategy outlined here is likely to be relevant to clinical management and genetic counseling.

## Competing interests

WK declares no conflict of interest. PKR and JHMK founded Cytognomix Inc. and are the inventors of US Patent Nos. 6,828,097, 7,014,997 and 7,734,424.

## Authors' contributions

PKR, JHMK: conceived and guided the project

PKR: coordinated the study

WK: performed bioinformatic analyses, SC and LC probe development, validation, FISH analyses, cell line culture and chromosome preparations

JHMK: provided the AS cell lines and validated cytogenetic preparations

WK, JHMK, PKR: wrote the manuscript. All authors have read and approved the final manuscript.

## Supplementary Material

Additional file 1 **Table S1 - SC and LC primers**. Optimized melting temperatures (Tm) and genomic positions (hg18) of the forward and reverse primer pairs. Click here for file

## References

[B1] Lupski JR Genomic disorders: structural features of the genome can lead to DNA rearrangements and human disease traits Trends Genet 1998 14 417 422 10.1016/S0168-9525(98)01555-8 9820031

[B2] Lupski JR Genomic disorders ten years on Genome Med 2009 1 42 10.1186/gm42 19439022 PMC2684663

[B3] Mefford HC Eichler EE Duplication hotspots, rare genomic disorders, and common disease Curr Opin Genet Dev 2009 19 196 204 10.1016/j.gde.2009.04.003 19477115 PMC2746670

[B4] Knoll JH Nicholls RD Magenis RE Graham JM Jr Lalande M Latt SA Angelman and Prader-Willi syndromes share a common chromosome 15 deletion but differ in parental origin of the deletion Am J Med Genet 1989 32 285 290 10.1002/ajmg.1320320235 2564739

[B5] Knoll JH Nicholls RD Magenis RE Glatt K Graham JM Jr Kaplan L Lalande M Angelman syndrome: three molecular classes identified with chromosome 15q11q13-specific DNA markers Am J Hum Genet 1990 47 149 154 1971993 PMC1683759

[B6] Nicholls RD Knepper JL Genome organization, function, and imprinting in Prader-Willi and Angelman syndromes Annu Rev Genomics Hum Genet 2001 2 153 175 10.1146/annurev.genom.2.1.153 11701647

[B7] Christian SL Fantes JA Mewborn SK Huang B Ledbetter DH Large genomic duplicons map to sites of instability in the Prader-Willi/Angelman syndrome chromosome region (15q11.2q13) Hum Mol Genet 1999 8 1025 1037 10.1093/hmg/8.6.1025 10332034

[B8] Amos-Landgraf JM Ji Y Gottlieb W Depinet T Wandstrat AE Cassidy SB Driscoll DJ Rogan PK Schwartz S Nicholls RD Chromosome breakage in the Prader-Willi and Angelman syndromes involves recombination between large, transcribed repeats at proximal and distal breakpoints Am J Hum Genet 1999 65 370 386 10.1086/302510 10417280 PMC1377936

[B9] Jiang YH Wauki K Liu Q Bressler J Pan Y Kashork CD Shaffer LG Beaudet AL Genomic analysis of the chromosome 15q11.2q13 Prader-Willi syndrome region and characterization of transcripts for GOLGA8E and WHCD1L1 from the proximal breakpoint region BMC Genomics 2008 9 50 10.1186/1471-2164-9-50 18226259 PMC2268926

[B10] Wang NJ Liu D Parokonny AS Schanen NC High-resolution molecular characterization of 15q11.2q13 rearrangements by array comparative genomic hybridization (array CGH) with detection of gene dosage Am J Hum Genet 2004 75 267 281 10.1086/422854 15197683 PMC1216061

[B11] Locke DP Segraves R Nicholls RD Schwartz S Pinkel D Albertson DG Eichler EE BAC microarray analysis of 15q11.2q13 rearrangements and the impact of segmental duplications J Med Genet 2004 41 175 182 10.1136/jmg.2003.013813 14985376 PMC1735707

[B12] Sahoo T Bacino CA German JR Shaw CA Bird LM Kimonis V Anselm I Waisbren S Beaudet AL Peters SU Identification of novel deletions of 15q11q13 in Angelman syndrome by array-CGH: molecular characterization and genotype-phenotype correlations Eur J Hum Genet 2007 15 943 949 10.1038/sj.ejhg.5201859 17522620

[B13] Butler MG Fischer W Kibiryeva N Bittel DC Array comparative genomic hybridization (aCGH) analysis in Prader-Willi syndrome Am J Med Genet 2008 146A 854 860 10.1002/ajmg.a.32249 PMC5438264 18266248

[B14] Nicholls RD Knepper JL Genome organization, function, and imprinting in Prader-Willi and Angelman syndromes Annu Rev Genomics Hum Genet 2001 2 153 175 10.1146/annurev.genom.2.1.153 11701647

[B15] Rogan PK Cazcarro PM Knoll JH Sequence-based design of single-copy genomic DNA probes for fluorescence in situ hybridization Genome Res 2001 11 1086 1094 10.1101/gr.171701 11381034 PMC311125

[B16] Knoll JH Rogan PK Sequence-based, in situ detection of chromosomal abnormalities at high resolution Am J Med Genet 2003 121A 245 257 10.1002/ajmg.a.20123 12923866

[B17] Yamada NA Rector LS Tsang P Carr E Scheffer A Sederberg MC Aston ME Ach RA Tsalenko A Sampas N Peter B Bruhn L Brothman AR Visualization of fine scale genomic structure by oligonucleotide-based high-resolution FISH Cytogenet Genome Res 2011 132 248 254 10.1159/000322717 21178330

[B18] Benson DA Karsch-Mizrachi I Lipman DJ Ostell J Sayers EW GenBank Nucleic Acids Res 2011 39 D32 37 10.1093/nar/gkq1079 21071399 PMC3013681

[B19] Miller DT Shen Y Weiss LA Korn J Anselm I Bridgemohan C Cox GF Dickinson H Gentile J Harris DJ Hegde V Hundley R Khwaja O Kothare S Luedke C Nasir R Poduri A Prasad K Raffalli P Reinhard A Smith SE Sobeih MM Soul JS Stoler J Takeoka M Tan WH Thakuria J Wolff R Yusupov R Gusella JF Microdeletion/duplication at 15q13.2q13.3 among individuals with features of autism and other neuropsychiatric disorders J Med Genet 2009 46 242 248 10.1136/jmg.2008.059907 18805830 PMC4090085

[B20] Blankenberg D Von Kuster G Coraor N Ananda G Lazarus R Mangan M Nekrutenko A Taylor J Ausubel F, Brent R, Kingston RE, Moore DD, Seidman JG, Smith JA, Struhl K Galaxy: a web-based genome analysis tool for experimentalists Curr Protoc Mol Biol 2010 Chapter 19 New Jersey: John Wiley & Sons, Inc. Unit 19.10.1-21 10.1002/0471142727.mb1910s89 PMC4264107 20069535

[B21] Itsara A Cooper GM Baker C Girirajan S Li J Absher D Krauss RM Myers RM Ridker PM Chasman DI Mefford H Ying P Nickerson DA Eichler EE Population analysis of large copy number variants and hotspots of human genetic disease Am J Hum Genet 2009 84 148 161 10.1016/j.ajhg.2008.12.014 19166990 PMC2668011

[B22] Rozen S Skaletsky H Primer3 on the WWW for general users and for biologist programmers Methods Mol Biol 2000 132 365 386 10547847 10.1385/1-59259-192-2:365

[B23] Cheng S Fockler C Barnes WM Higuchi R Effective amplification of long targets from cloned inserts and human genomic DNA Proc Natl Acad Sci 1994 91 5695 5699 10.1073/pnas.91.12.5695 8202550 PMC44063

[B24] Knoll JH Lichter P Haines JL, Korf BR, Morton CC, Seidman CE, Seidman JG, Smith DR In situ hybridization to metaphase chromosomes and interphase nuclei Curr Protoc Hum Genet 2005 Chapter 4 New Jersey: John Wiley & Sons, Inc. Unit 4.3 10.1002/0471142905.hg0403s45 18428378

[B25] Pujana MA Nadal M Gratacòs M Peral B Csiszar K González-Sarmiento R Sumoy L Estivill X Additional complexity on human chromosome 15q: identification of a set of newly recognized duplicons (LCR15) on 15q11.2q13,15q24, and 15q26 Genome Res 2001 11 98 111 10.1101/gr.155601 11156619 PMC311040

[B26] Ballif BC Hornor SA Jenkins E Madan-Khetarpal S Surti U Jackson KE Asamoah A Brock PL Gowans GC Conway RL Graham JM Jr Medne L Zackai EH Shaikh TH Geoghegan J Selzer RR Eis PS Bejjani BA Shaffer LG Discovery of a previously unrecognized microdeletion syndrome of 16p11.2-p12.2 Nat Genet 2007 39 1071 1073 10.1038/ng2107 17704777

[B27] Ji Y Rebert NA Joslin JM Higgins MJ Schultz RA Nicholls RD Structure of the highly conserved *HERC2 *gene and of multiple partially duplicated paralogs in human Genome Res 2000 10 319 329 10.1101/gr.10.3.319 10720573 PMC311424

[B28] Chai JH Locke DP Greally JM Knoll JH Ohta T Dunai J Yavor A Eichler EE Nicholls RD Identification of four highly conserved genes between breakpoint hotspots BP1 and BP2 of the Prader-Willi/Angelman syndromes deletion region that have undergone evolutionary transposition mediated by flanking duplicons Am J Hum Genet 2003 73 898 925 10.1086/378816 14508708 PMC1180611

[B29] Murphy SM Preble AM Patel UK O'Connell KL Dias DP Moritz M Agard D Stults JT Stearns T GCP5 and GCP6: two new members of the human gamma-tubulin complex Mol Biol Cell 2001 12 3340 3352 11694571 10.1091/mbc.12.11.3340 PMC60259

[B30] Schenck A Bardoni B Moro A Bagni C Mandel JL A highly conserved protein family interacting with the fragile × mental retardation protein (FMRP) and displaying selective interactions with FMRP-related proteins FXR1P and FXR2P Proc Natl Acad Sci 2001 98 8844 8849 10.1073/pnas.151231598 11438699 PMC37523

[B31] Morales J Hiesinger PR Schroeder AJ Kume K Verstreken P Jackson FR Nelson DL Hassan BA Drosophila fragile × protein, DFXR, regulates neuronal morphology and function in the brain Neuron 2002 34 961 972 10.1016/S0896-6273(02)00731-6 12086643

[B32] Rainer S Chai JH Tokarz D Nicholls RD Fink JK NIPA1 gene mutations cause autosomal dominant hereditary spastic paraplegia (SPG6) Am J Hum Genet 2003 73 967 971 10.1086/378817 14508710 PMC1180617

[B33] Apweiler R Martin MJ O'Donovan C Magrane M Alam-Faruque Y Antunes R Barrell D Bely B Bingley M Binns D Bower L Browne P Chan WM Dimmer E Eberhardt R Fedotov A Foulger R Garavelli J Huntley R Jacobsen J Kleen M Laiho K Leinonen R Legge D Lin Q Liu W Luo J Orchard S Patient S Poggioli D The Universal Protein Resource (UniProt) in 2010 Nucleic Acids Res 2010 38 D142 148 19843607 10.1093/nar/gkp846 PMC2808944

[B34] Lee ST Nicholls RD Bundey S Laxova R Musarella M Spritz RA Mutations of the P gene in oculocutaneous albinism, ocular albinism, and Prader-Willi syndrome plus albinism N Engl J Med 1994 330 529 534 10.1056/NEJM199402243300803 8302318

[B35] Duffy DL Montgomery GW Chen W Zhao ZZ Le L James MR Hayward NK Martin NG Sturm RA A three-single-nucleotide polymorphism haplotype in intron 1 of OCA2 explains most human eye-color variation Am J Hum Genet 2007 80 241 252 10.1086/510885 17236130 PMC1785344

[B36] Babatz TD Kumar RA Sudi J Dobyns WB Christian SL Copy number and sequence variants implicate APBA2 as an autism candidate gene Autism Res 2009 6 359 364 10.1002/aur.107 20029827

[B37] Battaglia A Novelli A Bernardini L Igliozzi R Parrini B Further characterization of the new microdeletion syndrome of 16p11.2-p12.2 Am J Med Genet A 2009 149A 1200 1204 10.1002/ajmg.a.32847 19449418

[B38] Szafranski P Schaaf CP Person RE Gibson IB Xia Z Mahadevan S Wiszniewska J Bacino CA Lalani S Potocki L Kang SH Patel A Cheung SW Probst FJ Graham BH Shinawi M Beaudet AL Stankiewicz P Structures and molecular mechanisms for common 15q13.3 microduplications involving CHRNA7: benign or pathological? Hum Mutat 2010 31 840 850 10.1002/humu.21284 20506139 PMC3162316

[B39] Hannes FD Sharp AJ Mefford HC de Ravel T Ruivenkamp CA Breuning MH Fryns JP Devriendt K Van Buggenhout G Vogels A Stewart H Hennekam RC Cooper GM Regan R Knight SJ Eichler EE Vermeesch JR Recurrent reciprocal deletions and duplications of 16p13.11: the deletion is a risk factor for MR/MCA while the duplication may be a rare benign variant J Med Genet 2009 46 223 232 10.1136/jmg.2007.055202 18550696 PMC2658752

[B40] Pani AM Hobart HH Morris CA Mervis CB Bray-Ward P Kimberley KW Rios CM Clark RC Gulbronson MD Gowans GC Gregg RG Genome rearrangements detected by SNP microarrays in individuals with intellectual disability referred with possible Williams syndrome PLoS One 2010 5 e12349 10.1371/journal.pone.0012349 20824207 PMC2930846

[B41] Ou Z Stankiewicz P Xia Z Breman AM Dawson B Wiszniewska J Szafranski P Cooper ML Rao M Shao L South ST Coleman K Fernhoff PM Deray MJ Rosengren S Roeder ER Enciso VB Chinault AC Patel A Kang SH Shaw CA Lupski JR Cheung SW Observation and prediction of recurrent human translocations mediated by NAHR between nonhomologous chromosomes Genome Res 2011 21 33 46 10.1101/gr.111609.110 21205869 PMC3012924

[B42] Shaikh TH Oligonucleotide arrays for high-resolution analysis of copy number alteration in mental retardation/multiple congenital anomalies Genet Med 2007 9 617 625 10.1097/GIM.0b013e318148bb81 17873650

[B43] Scherer SW Lee C Birney E Altshuler DM Eichler EE Carter NP Hurles ME Feuk L Challenges and standards in integrating surveys of structural variation Nat Genet 2007 39 Suppl 7 S7 15 17597783 10.1038/ng2093 PMC2698291

[B44] Neill NJ Torchia BS Bejjani BA Shaffer LG Ballif BC Comparative analysis of copy number detection by whole-genome BAC and oligonucleotide array CGH Mol Cytogenet 2010 3 11 10.1186/1755-8166-3-11 20587050 PMC2909945

[B45] Knijnenburg J Oberstein SA Frei K Lucas T Gijsbers AC Ruivenkamp CA Tanke HJ Szuhai K A homozygous deletion of a normal variation locus in a patient with hearing loss from non-consanguineous parents J Med Genet 2009 46 412 417 10.1136/jmg.2008.063685 19246478

[B46] Kato T Emi M Sato H Arawaka S Wada M Kawanami T Katagiri T Tsuburaya K Toyoshima I Tanaka F Sobue G Matsubara K Segmental copy-number gain within the region of isopentenyl diphosphate isomerase genes in sporadic amyotrophic lateral sclerosis Biochem Biophys Res Commun 2010 402 438 442 10.1016/j.bbrc.2010.10.056 20955688

[B47] Carter NP Methods and strategies for analyzing copy number variation using DNA microarrays Nat Genet 2007 39 Suppl 7 S16 21 17597776 10.1038/ng2028 PMC2697494

[B48] Alkan C Kidd JM Marques-Bonet T Aksay G Antonacci F Hormozdiari F Kitzman JO Baker C Malig M Mutlu O Sahinalp SC Gibbs RA Eichler EE Personalized copy number and segmental duplication maps using next-generation sequencing Nat Genet 2009 41 1061 1067 10.1038/ng.437 19718026 PMC2875196

